# A Distance Boundary with Virtual Nodes for the Weighted Centroid Localization Algorithm†

**DOI:** 10.3390/s18041054

**Published:** 2018-04-01

**Authors:** Kwang-Yul Kim, Yoan Shin

**Affiliations:** School of Electronic Engineering, Soongsil University, Seoul 06978, Korea; kky1117@ssu.ac.kr

**Keywords:** wireless sensor network, weighted centroid localization, distance boundary, intersection threshold, distribution map, test node

## Abstract

In wireless sensor networks, accurate location information is important for precise tracking of targets. In order to satisfy hardware installation cost and localization accuracy requirements, a weighted centroid localization (WCL) algorithm, which is considered a promising localization algorithm, was introduced. In our previous research, we proposed a test node-based WCL algorithm using a distance boundary to improve the localization accuracy in the corner and side areas. The proposed algorithm estimates the target location by averaging the test node locations that exactly match with the number of anchor nodes in the distribution map. However, since the received signal strength has large variability in real channel conditions, the number of anchor nodes is not exactly matched and the localization accuracy may deteriorate. Thus, we propose an intersection threshold to compensate for the localization accuracy in this paper. The simulation results show that the proposed test node-based WCL algorithm provides higher-precision location information than the conventional WCL algorithm in entire areas, with a reduced number of physical anchor nodes. Moreover, we show that the localization accuracy is improved by using the intersection threshold when considering small-scale fading channel conditions.

## 1. Introduction

Wireless sensor networks (WSNs) have been rapidly developed, and show promise for a variety of applications in next-generation networks. One of the main purposes of WSNs is real-time localization to track and search for moving targets. Thus, the search for a cost-effective localization method which guarantees high precision in real time is required in various WSN applications such as public safety, industrial applications, and military surveillance [1,2]. In order to guarantee high-precision localization, range-based localization methods have been proposed [3,4]. In range-based localization methods, the system uses extra equipment that estimates the location of an unknown node by measuring the distance or the angle between a number of anchor nodes and the unknown node. The representative schemes for the range-based localization are time of arrival (ToA), time difference of arrival (TDoA), angle of arrival (AoA), and received signal strength (RSS). However, since these schemes require a high-speed analog-to-digital converter because of their high complexity as well as extra equipment for measuring distances, they are not cost-effective schemes. On the other hand, since the range-free localization method estimates the location without the measurement of the distance or the angle, the method can reduce system complexity and create a cost-effective localization system which guarantees the real-time operation more easily. The representative schemes for the range-free localization are centroid localization (CL), the approximate point-in-triangulation test (APIT), knowledge-based positioning, distance vector (DV)-hop, and range-free AoA [3,4,5]. Since the sensor node must consider power consumption as well as system complexity, almost all of the localization algorithms will require a simple structure and a rapid processing speed. Thus, the CL algorithm is considered as an effective method to meet the requirements of the localization system in WSNs among the various localization algorithms.

Although the CL algorithm has the advantage of making localization systems cost-effective, it has the disadvantage of reducing the location accuracy. Recently, a weighted CL (WCL) method which combines the CL with the range-based localization method has been proposed to improve the location accuracy by giving the weight according to the difference in distance [6,7,8]. Since the localization operation is simply performed by using only the anchor node location (as in the case of the CL), the WCL algorithm is also effective in terms of the processing speed and resource utilization. Most range-based indoor localization research measures utilize the RSS to calculate the distance between the unknown moving node and a number of anchor nodes. This is mainly due to the fact that the RSS measurements can be obtained with minimal effort and do not require any extra equipment (as occurs with the range-based localization methods), resulting in remarkable savings in the cost and energy consumption of sensor nodes. Moreover, most transceivers in WSNs have built-in RSS indicators (RSSIs) which provide the RSS measurement value depending on their infrastructure without any extra cost.

However, the localization accuracy of the WCL algorithm deteriorates significantly at the corners and sides of the square space due to the centroid property [9]. One simple approach to improve the localization accuracy for these areas is to increase the number of anchor nodes. However, the number of anchor nodes is limited because of the system restrictions such as hardware installation cost and resource (frequency, energy) consumption. In order to overcome the limitations, many authors have used virtual concepts [10,11,12]. In [10], the authors proposed a novel indoor localization approach named virtual reference elimination (VIRE). This method calculates the RSS values of the virtual reference tags using a linear interpolation algorithm. In [11], the authors proposed an improved CL combined with the VIRE system using the RSS of the virtual reference tags. However, this algorithm considered only a path-loss model without small-scale fading. In [12], the authors proposed a novel algorithm with the small extra logical overhead which used the shortest-hop path scheme to upgrade the virtual anchor nodes, whereas the number of physical anchor nodes was kept the same. However, this algorithm was studied in another range-free localization method: the DV-hop. Moreover, in [13,14], we have already studied the WCL using a test node similar to the conventional virtual anchor node. However, in these previous papers, we did not consider the inconsistency of the number of anchor nodes in a real channel condition nor did we evaluate the localization accuracy of the deployment of test nodes. Moreover, since the RSS has large variability due to the degrading effects of reflection, shadowing, and fading in indoor wireless channel environments [15], the test node-based WCL (T-WCL) algorithm may reduce the localization accuracy because the numbers of anchor nodes in the distance boundary of the unknown node and in the test nodes are mismatched.

In this paper, we propose the T-WCL algorithm with an intersection threshold to improve the localization accuracy. The contributions of this paper are summarized as follows. First, the test node as the virtual node can be considered as a means of improving the localization accuracy and reducing the hardware installation cost of the conventional WCL (C-WCL) algorithm [9]. Second, in order to evaluate the performance in a situation similar to real channel conditions, we consider a path-loss channel model with log-normal shadowing. Third, the intersection threshold can be considered as an important factor to improve the localization accuracy for the WCL algorithm in small-scale fading channels.

The rest of the paper is organized as follows. The characterization of the indoor propagation channel is described in Section 2, which deals with the adopted channel model and the conventional WCL algorithm. Section 3 presents our proposed test node-based WCL algorithm. Section 4 evaluates the algorithmic performance of each WCL algorithm by computer simulations. Finally, conclusions are given in Section 5.

## 2. System Model

We consider a WSN with *N* anchor nodes located in a square area of side length *S*, and a moving unknown node located within the area at $L_{p}={\left[x_{p},y_{p}\right]}^{T}$, where ${\left[\cdot \right]}^{T}$ denotes the transpose operation. The location of the *i*-th anchor node is defined as $L_{i}={\left[x_{i},y_{i}\right]}^{T}$, i=1,⋯,N. Then, the WCL estimates the unknown node location ${\hat{L}}_{p}$ as

(1) $${\hat{L}}_{p}=\frac{\sum_{i=1}^{N}w_{i}L_{i}}{\sum_{i=1}^{N}w_{i}},$$

where $w_{i}$ is the weighting coefficient which implies the influence deciding the extent of the unknown node close to the *i*-th anchor node. Most of the localization algorithms require the distance measurement to estimate the location of the unknown moving node accurately. As mentioned in Section 1, one of the efficient methods for measuring the distance is the RSS method. There are three representative wireless propagation models for the RSS method: the free-space model, the two-ray ground reflection model, and the shadowing model. In Friis’ free-space model, the detected RSS decreases quadratically with the distance to the transmitter which is given as [16]

(2) $$\mathrm{RSS}\left(d_{i}\right)=P_{t}G_{t}G_{r}{\left(\frac{\lambda }{4\pi d_{i}}\right)}^{2},$$

where $P_{t}$ is the transmitting power at the anchor node, $G_{t}$ is the gain at the anchor node, $G_{r}$ is the gain at the unknown node, λ is the wavelength, and $d_{i}={∥L_{i}-L_{p}∥}_{2}$ is the distance between the *i*-th anchor node location $L_{i}$ and the unknown node location $L_{p}$. However, since the RSS at a certain distance is a random variant due to the multi-path fading effects in real channel conditions, we assume that the RSS follows the log-normal shadowing path-loss model. The log-normal shadowing path-loss model is expressed in decibels as [16]

(3) $$\mathrm{RSS}\left(d_{i}\right)=P_{t}+K-10\eta \log\left(\frac{d_{i}}{d_{0}}\right)+\psi .$$

Here, $d_{0}$ is the reference distance, *K* is the attenuation factor at the reference distance $d_{0}$, η is the path-loss exponent (commonly 2–4 for indoors), and ψ is a zero-mean Gaussian random variable with variance 1. Usually, $w_{i}$ is defined as a function of estimated distance and the relation is inversely proportional to estimated distance as $1/d_{i}$. To make the farther anchor node with lower weights, *g* is used as the power of distance as $w=1/{\left(d_{i}\right)}^{g}$. However, the RSS has some fluctuation; the accuracy of localization is significantly improved when the weight of the anchor node is set to 0 or 1 instead of $1/d_{i}$ [9]. Thus, in order to improve the performance of the WCL algorithm, we assume that the anchor nodes within the circle have the same weight as 1.

**Figure 1:**
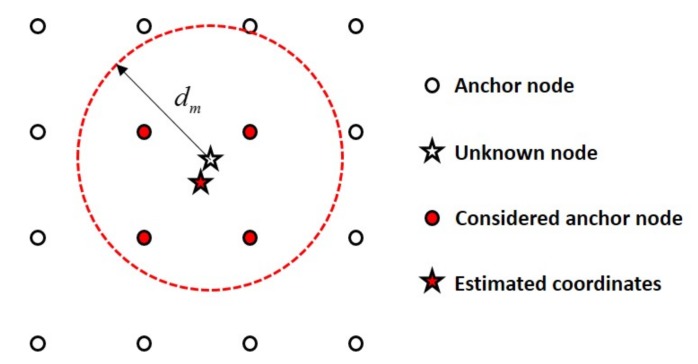
Operation of the conventional weighted centroid localization (C-WCL) algorithm with a distance boundary [9,14].

Figure 1 shows the operation of the C-WCL algorithm with a distance boundary. In Figure 1, a distance boundary $d_{m}$ from the unknown node becomes a radius of the circle and is indicated as a threshold to choose the considered anchor nodes. Generally, in order to estimate the unknown node, the C-WCL algorithm considers the anchor nodes within the distance boundary only and averages the locations of these anchor nodes. Thus, the distance boundary $d_{m}$ plays an important role in C-WCL, improving the localization accuracy. Then, the localization error is defined as $\xi ≜{\hat{L}}_{p}-L_{p}=\left[{\hat{X}}_{p}-x_{p},{\hat{Y}}_{p}-y_{p}\right]T$, where ${\hat{X}}_{p}$ and ${\hat{Y}}_{p}$ are the one-dimensional location estimates along the *x*-axis and *y*-axis, respectively. Finally, the distance error is given by

(4) $$\xi ≜\sqrt{{\left({\hat{X}}_{p}-x_{p}\right)}^{2}+{\left({\hat{Y}}_{p}-y_{p}\right)}^{2}}={∥\xi ∥}_{2}.$$

## 3. The Proposed Test Node-Based WCL Algorithm

In order to improve the localization accuracy in the corner and edge areas and solve the mismatch problem due to the small-scale fading channel, we propose a T-WCL algorithm with an intersection threshold. The proposed T-WCL algorithm consists of five steps. The detailed procedures are introduced below.

### 3.1. Test Node Generation

In the first step, the proposed T-WCL algorithm deploys the test nodes between the anchor nodes during the off-line phase. Since the test node is a virtual node made from the algorithm, we can systematically calculate the exact distance between each virtual test node and a number of real anchor nodes. We consider a WSN with *M* test nodes and assume that there are *M* sensors deployed uniformly in an *n* by *k* sensor field grid.

### 3.2. Distribution Map Construction

In the second step, after generating the test nodes, the T-WCL algorithm constructs a distribution map. The main operation of the distribution map is to store the number of anchor nodes $C_{test}\left(T_{j}\right)$ and their coordinates $L_{i}$ within the distance boundary $d_{m}$ made from each test node. This process is conducted by comparing the distance between each test node and a number of anchor nodes with distance boundary $d_{m}$ virtually. For the *j*-th test node location $T_{j}={\left[x_{j},y_{j}\right]}^{T}$, where j=1,⋯,M, we denote the Euclidean distance between the *i*-th anchor node location $L_{i}$ and *j*-th test node location $T_{j}$ as $d\left(L_{i},T_{j}\right)=\sqrt{{\left(x_{i}-x_{j}\right)}^{2}+{\left(y_{i}-y_{j}\right)}^{2}}$. Then, the algorithm counts the number of anchor nodes $C_{test}\left(T_{j}\right)$ within the distance boundary of *j*-th test node as

(5) $$C_{test}\left(T_{j}\right)=\sum_{i=1}^{N}a_{i},\;a_{i}=\left\{\begin{array}{cc} 1, & \mathrm{if}\;d\left(L_{i},T_{j}\right)<d_{m}\\ 0, & \mathrm{otherwise} \end{array}\right..$$

Finally, we can construct the distribution map $\mathrm{DM}=\left\{{\left[T_{1},\cdots ,T_{M}\right]}^{T},{\left[C_{test}\left(T_{1}\right),\cdots ,C_{test}\left(T_{M}\right)\right]}^{T}\right\}$. Here, the distribution map DM contains the coordinates $L_{i}$ of considered anchor nodes corresponding to $C_{test}\left(T_{j}\right)$.

### 3.3. Test Node Detection

The key point of the proposed T-WCL algorithm to estimate the location of the unknown node is to compare the difference in the number of considered anchor nodes. In the on-line phase, the unknown node receives the RSS from specific anchor nodes in real time. Then, the unknown node transmits the RSS and anchor identification (ID) to the localization system. In the system, the algorithm calculates the distance $d_{r}$ from the RSS by using Equation (3) except ψ and compares each calculated distance with the distance boundary $d_{m}$ to obtain the number of considered anchor nodes. The algorithm counts the number of considered anchor nodes $C_{unknown}\left(L_{p}\right)$ at the unknown node as

(6) $$C_{unknown}\left(L_{p}\right)=\sum_{i=1}^{N}a_{i},\;a_{i}=\left\{\begin{array}{cc} 1, & \mathrm{if}\;d_{r}<d_{m}\\ 0, & \mathrm{otherwise} \end{array}\right..$$

Then, the proposed T-WCL algorithm detects the matched test node that satisfies the condition $C_{test}\left(T_{j}\right)=C_{unknown}\left(L_{p}\right)$. For example, if the number of anchor nodes $C_{unknown}\left(L_{p}\right)$ at the unknown node is 5, the algorithm compares 5 with the number of considered anchor nodes $C_{test}\left(T_{j}\right)$ at the test node in the distribution map DM.

### 3.4. Intersection

In real channel conditions, there are many identical numbers that exist in other regions. Thus, in this step, the algorithm compares the coordinates $L_{i}$ of the anchor nodes obtained from the unknown node with the coordinates of the anchor nodes obtained from each test node within the area. However, since the RSS has large variability in real channel conditions, the number of anchor nodes may not be exactly matched and the localization accuracy deteriorates. Thus, we consider the intersection threshold $A_{th}$, and then the comparing factor is expressed as

(7) $$T_{th}=C_{test}\left(T_{j}\right)-A_{th}.$$

The test nodes must be within the range $T_{th}⩽C_{intersect}\left(T_{j}\right)⩽C_{test}\left(T_{j}\right)$ to be assigned the weights, where $C_{intersect}\left(T_{j}\right)$ is the number of intersected coordinates $L_{i}$ of considered anchor nodes at the *j*-th test node.

### 3.5. Estimation

The last step is the location estimation of the unknown node by allocating the weight $w_{j}$. Finally, the algorithm estimates the coordinates of the unknown node as

(8) $${\hat{L}}_{p}=\frac{\sum_{j=1}^{L}w_{j}T_{j}}{\sum_{j=1}^{L}w_{j}}.$$

Here, *L* denotes the number of intersected test nodes (L<M). As this is identical to (1), it is assumed that the test nodes only match with the number and the coordinates of the anchor nodes obtained from the unknown node, which have a weight equal to 1.

## 4. Simulation Results

### 4.1. Simulation Environments

In order to evaluate the localization accuracy of the proposed T-WCL algorithm according to the distance boundary $d_{m}$ and the number of anchor nodes, we performed the simulations using MATLAB. For the simulations, we considered a 90×90 (m${}^{2}$) ideal square space, and the anchor nodes were distributed on the same horizontal area with the exact coordinates. The test nodes generated virtually by the proposed T-WCL algorithm were deployed equally on the above square space. The distribution map contained the information (number and coordinates of considered anchor nodes) after calculating the coordinates of the anchor nodes in the circle and comparing the distance boundary $d_{m}$ from each test node. The unknown nodes were randomly disposed of, and transmitted the RSS and anchor ID to the localization system. We assumed that all physical nodes were equipped with one isotropic antenna, and the receiver sensitivity was −110 (dBm). The path-loss model with log-normal shadowing was considered as the localization channel model. In this paper, the path-loss exponent η was considered as 4 for the indoor environment, and the shadowing effect ψ was considered as a log-normal random variable with a mean of 0 and the standard deviation of 4 (dB). Finally, we changed the distance boundary $d_{m}$, the anchor node deployment interval $A_{d}$, the test node deployment interval $T_{d}$, and the intersection threshold $A_{th}$ to evaluate each WCL algorithm. We calculated the mean distance error (MDE) which is the average distance between the actual location $L_{p}$ of unknown nodes and their estimated location ${\hat{L}}_{p}$ to evaluate the localization accuracy for each WCL algorithm.

### 4.2. Effects of Test Node

The test node was introduced to improve the localization accuracy of the corner and side areas. In order to compare the localization accuracy of each area, we classified the WSN areas into one center region, four corner regions, and four side regions. Here, the size of the center region was 70×70 (m${}^{2}$), the sizes of the four corner regions were each 10×10 (m${}^{2}$), and the sizes of the four side regions were each 10×70 (m${}^{2}$). We assumed the anchor node deployment interval $A_{d}=10$ (m), the test node deployment interval $T_{d}=1$ (m), and the distance boundary $d_{m}=30$ (m). Each simulation result is averaged over 10,000 runs.

**Figure 2:**
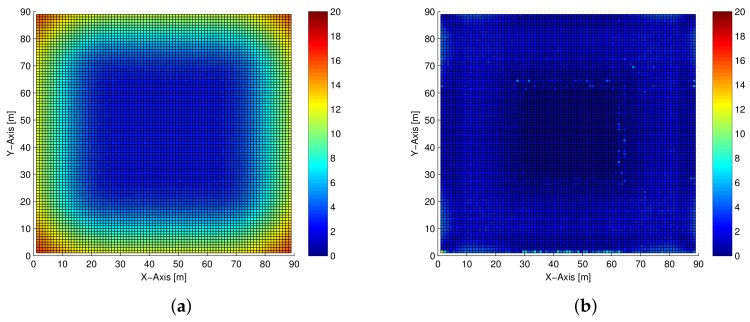
Top view of the distance error using each algorithm. (**a**) C-WCL; (**b**) test node-based WLC (T-WCL).

Figure 2 shows the distance error of each region by coloring with ranges from 0 to 20 (m). From Figure 2a, in the C-WCL algorithm case, we can observe that the distance errors at the corner and side areas were relatively larger than at the center area. The reason is that the corner and side areas have the insufficiently symmetric coordinates of the anchor nodes, that differ from the center area. On the other hand, in Figure 2b we can observe in the proposed T-WCL algorithm that the localization accuracy obtained at the corner and side areas was the same as at the center area, by densely deploying the virtual test nodes.

### 4.3. Effects of Distance Boundary

The distance boundary $d_{m}$ is an important factor for improving the localization accuracy and reducing the hardware installation cost of the WCL-based localization systems. In particular, a longer distance boundary $d_{m}$ is required to transmit the localization signal in the sparsely deployed anchor nodes without any performance degradation. In order to observe the effects of the distance boundary $d_{m}$ in each WCL algorithm, we calculate the MDE according to the distance boundary $d_{m}$. In order to simulate, we assumed the anchor node deployment interval $A_{d}=10$ (m), the test node deployment interval $T_{d}=1$ (m), and the distance boundary $d_{m}$ ranging from 10 to 30 (m). Each simulation result is averaged over 10,000 runs.

**Figure 3:**
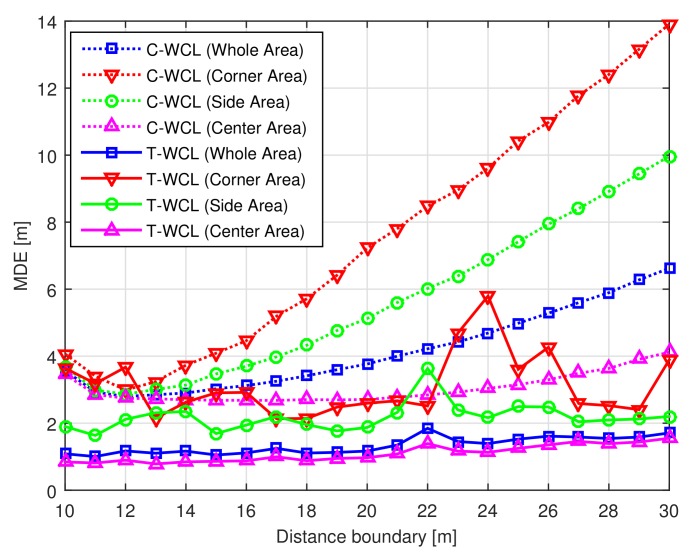
Mean distance error (MDE) performance of each area according to distance boundary.

Figure 3 shows the MDE performance of each area according to the distance boundary $d_{m}$. From Figure 3, we can observe that the MDE of the C-WCL algorithm is deteriorated by increasing the distance boundary $d_{m}$ due to significant localization errors in the corner and side areas. However, it is obvious that the proposed T-WCL algorithm which uses the virtual test nodes always outperforms the C-WCL, even though we increase the distance boundary $d_{m}$. Moreover, the proposed T-WCL algorithm with the distance boundary $d_{m}=30$ (m) can improve the localization accuracy by almost 74.2% compared to the C-WCL algorithm for the whole area.

### 4.4. Effects of Anchor Node Deployment Interval

In WSNs, the density of physical anchor nodes has significant effects on the localization accuracy. However, the high density of physical anchor nodes implies the additional costs of hardware installation and maintenance [9]. In order to make WSNs cost effective, we have to consider the trade-off between the localization accuracy and the hardware installation cost. Since the test node is also introduced to reduce the installation cost of the indoor localization systems, we can estimate that if we can reduce the physical anchor nodes and replace them with the virtual test nodes, the localization systems can be improved significantly in terms of the localization performance as well as the system installation and maintenance costs. In order to evaluate the localization accuracy and cost effectiveness of the proposed T-WCL algorithm, we calculate the MDE according to the distance boundary $d_{m}$ with the anchor node deployment interval.

**Figure 4:**
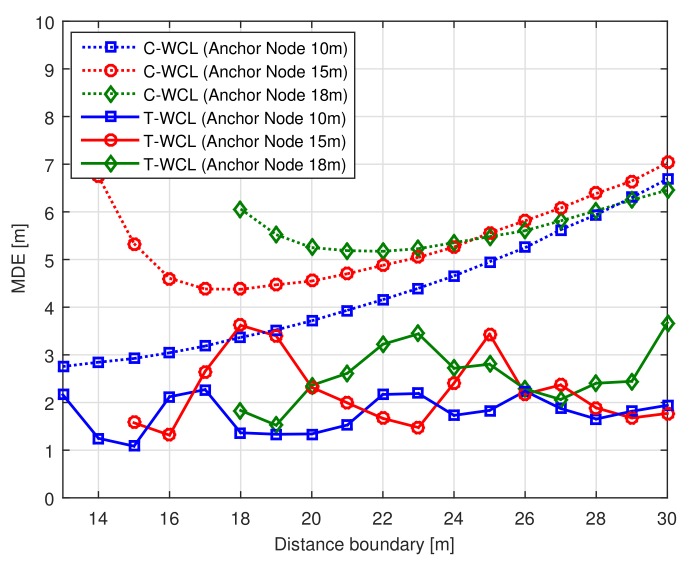
MDE performance according to the distance boundary with the anchor node deployment interval.

We assumed the physical anchor node deployment interval $A_{d}$ to be 10, 15, 18 (m; the number of anchor nodes was 100, 49 and 36). The virtual test node deployment interval was assumed to be $T_{d}$ = 1 (m; the number of test nodes was 8281). The latter is an interval to improve the localization accuracy by densely deploying the virtual nodes rather than the physical nodes. Note that the physical nodes were sparsely distributed with the regular intervals, and the unknown node was located between these physical nodes. Figure 4 shows the MDE performance according to the distance boundary with anchor node deployment interval $A_{d}$. In Figure 4, we can observe that the MDE performance of the C-WCL algorithm is deteriorated by increasing the anchor node deployment interval $A_{d}$ according to the distance boundary $d_{m}$. However, in spite of the lower-density condition of the anchor nodes, the proposed T-WCL algorithm can improve the localization accuracy. From the simulation results, we can conclude that the proposed T-WCL algorithm makes the localization system very cost effective by reducing the number of physical anchor nodes by up to 64%.

### 4.5. Effects of Test Node Deployment Interval

**Figure 5:**
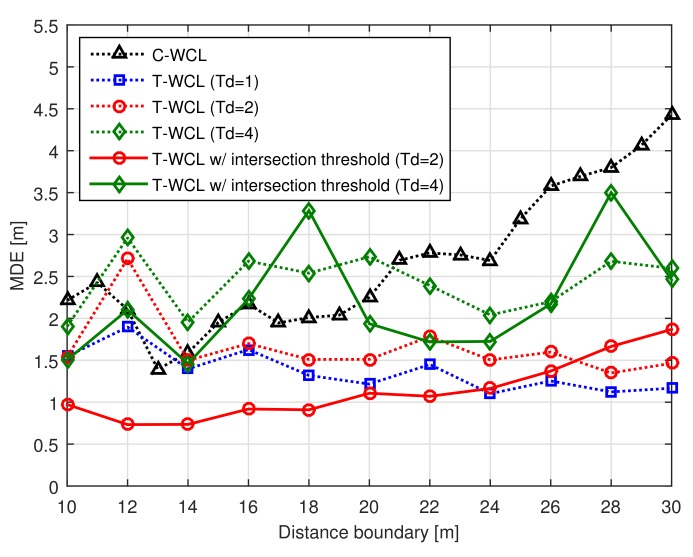
MDE performance according to the distance boundary with the test node deployment interval.

Figure 5 shows the MDE performance according to the distance boundary with the test node deployment interval $T_{d}$. From Figure 5, we can observe that the proposed T-WCL algorithm using the intersection threshold $A_{th}$ can improve the localization accuracy in the real environment. In particular, in the case of the test node deployment interval $T_{d}$ = 2 (m), the localization accuracy was improved, in spite of the number of test nodes being reduced by almost 73.2%. Therefore, we can conclude that the complexity can be reduced considerably and the localization accuracy improved. However, in the case of the test node deployment interval $T_{d}$ = 4 (m), since the number of test nodes was reduced by almost 92.4%, the localization accuracy was reduced. The reason is that the proposed T-WCL algorithm estimates the unknown node location by averaging the location of the test nodes.

### 4.6. Effects of Intersection Threshold

**Figure 6:**
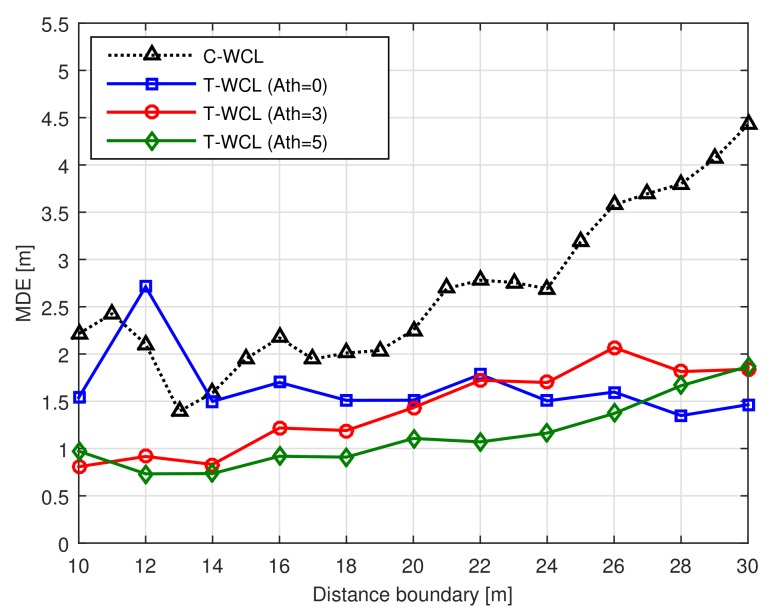
MDE performance according to the distance boundary with the intersection threshold.

Figure 6 shows the MDE performance according to the distance boundary with the intersection threshold $A_{th}$ at $T_{d}=2$ (m). From Figure 6, we can observe that the proposed T-WCL algorithm with large intersection threshold $A_{th}$ can improve the localization accuracy in the real environment. However, when the intersection threshold $A_{th}$ is set too low, a weighting value is given to a test node with a low correlation, so that the localization error becomes large. Finally, we can conclude that the proposed intersection threshold $A_{th}$ can improve the location accuracy for the variation of the RSS value in real channel conditions.

## 5. Conclusions

In WSNs, the C-WCL algorithm exploiting the distance boundary has been considered as an important algorithm for improving localization accuracy and system complexity. However, since the corner and side areas do not have enough symmetrically-placed anchor nodes (unlike the center area), the localization accuracy deteriorates in those areas of the square space. In order to overcome the drawbacks of the C-WCL algorithm, we proposed the T-WCL algorithm. From the simulation results, the proposed T-WCL algorithm guarantees the equal localization accuracy in each area and significantly improves the localization accuracy. We can observe that the proposed T-WCL algorithm can construct cost-effective localization systems by reducing the number of real anchor nodes. Furthermore, we can conclude that the localization accuracy is improved by using the intersection threshold in small-scale fading channels.
